# Clinical implications of next‐generation sequencing‐based panel tests for malignant ovarian tumors

**DOI:** 10.1002/cam4.3383

**Published:** 2020-08-19

**Authors:** Keiko Saotome, Tatsuyuki Chiyoda, Eriko Aimono, Kohei Nakamura, Shigeki Tanishima, Sachio Nohara, Chihiro Okada, Hideyuki Hayashi, Yuka Kuroda, Hiroyuki Nomura, Nobuyuki Susumu, Takashi Iwata, Wataru Yamagami, Fumio Kataoka, Hiroshi Nishihara, Daisuke Aoki

**Affiliations:** ^1^ Department of Obstetrics and Gynecology Keio University School of Medicine Tokyo Japan; ^2^ Genomics Unit Keio Cancer Center Keio University School of Medicine Tokyo Japan; ^3^ Department of Biomedical Informatics Development Mitsubishi Space Software Co., Ltd Amagasaki Japan; ^4^ Department of Obstetrics and Gynecology School of Medicine Fujita Health University Toyoake Japan; ^5^ Department of Obstetrics and Gynecology International University of Health and Welfare, School of Medicine Narita Japan; ^6^ Present address: Department of Obstetrics and Gynecology Eiju General Hospital Tokyo Japan; ^7^ Present address: Department of Obstetrics and Gynecology International University of Health and Welfare, School of Medicine Narita Japan

**Keywords:** Actionable gene alteration, Clinical sequencing, Druggable gene alteration, Ovarian cancer, Precision medicine

## Abstract

Precision medicine based on cancer genomics is being applied in clinical practice. However, patients do not always derive benefits from genomic testing. Here, we performed targeted amplicon exome sequencing‐based panel tests, including 160 cancer‐related genes (PleSSision‐160), on 88 malignant ovarian tumors (high‐grade serous carcinoma, 27; endometrioid carcinoma, 15; clear cell carcinoma, 30; mucinous carcinoma, 6; undifferentiated carcinoma, 4; and others, 6 (immature teratoma, 1; carcinosarcoma, 3; squamous cell carcinoma, 1; and mixed, 1)), to assess treatment strategies and useful biomarkers for malignant ovarian tumors. Overall, actionable gene variants were found in 90.9%, and druggable gene variants were found in 40.9% of the cases. Actionable *BRCA1* and *BRCA2* variants were found in 4.5% of each of the cases. *ERBB2* amplification was found in 33.3% of mucinous carcinoma cases. Druggable hypermutation/ultramutation (tumor mutation burden ≥ 10 SNVs/Mbp) was found in 7.4% of high‐grade serous carcinoma, 46.7% of endometrioid carcinoma, 10% of clear cell carcinoma, 0% of mucinous carcinoma, 25% of undifferentiated carcinoma, and 33.3% of the other cancer cases. Copy number alterations were significantly higher in high‐grade serous carcinoma (*P* < .005) than in other histologic subtypes; some clear cell carcinoma showed high copy number alterations that were correlated with advanced stage (*P* < .05) and worse survival (*P* < .01). A high count of copy number alteration was associated with worse survival in all malignant ovarian tumors (*P* < .05). Our study shows that targeted agents can be detected in approximately 40% of malignant ovarian tumors via multigene panel testing, and copy number alteration count can be a useful marker to help assess risks in malignant ovarian tumor patients.

## Graphical abstract

Next‐generation sequencing‐based panel tests on 88 malignant ovarian tumors revealed that targeted agents can be detected in approximately 40% of malignant ovarian tumors. Copy number alteration count was found to be a useful marker to help assess risks in malignant ovarian tumor patients.

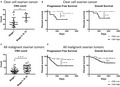

## 1 INTRODUCTION

Ovarian cancer is a devastating disease with a 5‐year survival rate of approximately 40%, and this survival rate has not improved in the past 30 years.^1^ Approved molecular targeted drugs include antivascular endothelial growth factor monoclonal antibody, bevacizumab, and poly (ADP‐ribose) polymerase (PARP) inhibitors; olaparib, niraparib, and rucaparib. Currently, there is no established biomarker for bevacizumab, which can prolong progression‐free survival (PFS) for several months; however, cancer usually regrows after or during bevacizumab maintenance therapy. PARP inhibitors are effective in ovarian cancers with homologous recombination deficiency (HRD), 40% of which are attributed to *BRCA1*/*BRCA2* germline or somatic variants. Novel biomarker‐based molecular target drugs are needed for high‐grade serous carcinoma (HGSC) without HRD, and other ovarian cancer histotypes, including endometrioid carcinoma (EC), clear cell carcinoma (CCC), and mucinous carcinoma (MC).

Next‐generation sequencing‐based genomic testing is currently used in clinical settings. MSK‐IMPACT and Foundation One CDx were approved by the US Food and Drug Administration. In Japan, Foundation One CDx and NCC OncoPanel received insurance coverage approval for advanced cancer patients who had progressive diseases after standard of care. However, only a low percentage of the patients undergo therapeutics recommended by genomic testing. It is reported that of the 59.4%‐85% of patients with actionable gene aberrations, only 13.3%‐24% received molecular targeted therapy.^2^, ^3^ We previously reported that only 10% of pancreatic cancer patients who underwent a targeted amplicon exome sequencing for 160 cancer‐related genes (PleSSision‐160) could be treated with therapeutic agents based on the results of genomic testing.^4^ Clinical sequencing for ovarian cancer is necessary due to its poor prognosis; however, reports regarding ovarian cancer are scarce.^2^, ^5^, ^6^ Currently, there is no comprehensive report on genomic testing of malignant ovarian tumors. Here, we report the results of PleSSision‐160 in malignant ovarian tumors, including four major histologic subtypes and rare tumor histologies, such as immature teratoma and carcinosarcoma.

## 2 MATERIALS AND METHODS

### 2.1 Patient population

This study included patients with malignant ovarian neoplasm who underwent surgery from January 2012 to September 2017 at the Keio University Hospital. The study protocol was approved by the ethics committee of Keio University (2007081, 20180214). All study participants provided informed consent. This study was performed following all relevant guidelines and regulations.

### 2.2 Next‐generation sequencing‐based multiplex gene assay (PleSSision‐160)

Frozen tissue samples collected from the patients who underwent surgeries were fixed using the PAXgene Tissue System (QIAGEN, Germantown, MD, USA) and embedded in paraffin. A pathologist evaluated tumor cell content by staining slides with hematoxylin and eosin, and macro‐dissected, if necessary. Genomic DNA was extracted and purified, and the DNA quality was checked based on the DNA integrity number (DIN) score calculated using the Agilent 2000 TapeStation (Agilent Technologies, Waldbronn, Germany). Thereafter, DNA libraries were prepared for genome sequencing of DNA with a DIN score over 3.1. Subsequently, we performed targeted amplicon exome sequencing for 160 cancer‐related genes (Table S1) using the Illumina MiSeq sequencing platform (Illumina, San Diego, CA, USA). Genome annotation and curation for analyzing the sequencing data were performed using an original bioinformatics pipeline called GenomeJack (Mitsubishi Space Software, Tokyo, Japan). We identified cancer‐specific somatic gene alterations, such as single nucleotide variations (SNVs), insertions/deletions (indels), and copy number variations (CNVs), as previously described.^7^ Furthermore, the tumor mutation burden (TMB) and copy number alterations (CNAs) were calculated using these data. The detailed method used for counting CNA is as follows; for calculating the baseline data used for count correction per amplicon, the number of leads sequenced in each of the 160‐panel amplicon probe design domains was counted to calculate the reads per million (RPM) value (ie, the number of leads per one million sequence leads). Then, the RPM coefficient of variation (CV), mean, and median value per amplicon in at least 100 formalin‐fixed paraffin‐embedded (FFPE) samples were counted. Thereafter, the RPM median of the amplicons with CV < 0.32 and mean > 10 was set as the baseline. The number of leads sequenced in each of the 160‐panel amplicon probe design domains was counted in the sample to calculate the copy number (CN) of each sample (calculating CN value to calculate the RPM value). Then, the baseline ratio {log2 ratio [= log2 (sample RPM/baseline RPM. median)]} in the amplicons that satisfied the conditions of CV < 0.32 and mean > 10 were counted, and the overall SD and median value of log2 ratio for each gene were calculated. The genes with a log2 ratio median value exceeding SD were categorized as amplification (amp)‐like, and those exceeding 2SD were categorized as amp. In addition, the genes with a log2 ratio median value below ‐SD were categorized as loss‐like, and those below −2SD were categorized as loss. As a control, 50 genomic DNA samples were used to normalize the read depth per amplicon. Only amplicons with CV of the depth ≤ 1.5 were used. CNA was calculated for the genes that had more than six amplicons, and it was measured as the median value of each amplicon per gene. The analysis reports were discussed and reviewed in a conference of genome experts consisting of medical oncologists, molecular oncologists, pathologists, medical geneticists, clinical laboratory technicians, and bioinformaticians. Hypermutation was defined as ≥ 10 SNVs/Mbp, and ultramutation was defined as ≥ 100 SNVs/Mbp.

### 2.3 Statistical analyses

Statistical analyses were performed with GraphPad Prism 7 (GraphPad Software, Inc, San Diego, CA, USA).

## 3 RESULTS

### 3.1 Patient characteristics

Sequencing was performed for 88 ovarian malignant neoplasms (HGSC: 27, EC: 15, CCC: 30, MC: 6, undifferentiated carcinoma (UC): 4, and others: 6 (immature teratoma: 1, carcinosarcoma: 3, squamous cell carcinoma: 1, and mixed (serous + clear cell): 1)). Overall, the median age was 55 (range: 28‐86), clinical stages were I: 41, II: 6, III: 32, IV: 9, and the tumors with recurrence were present in 30 cases (34.1%). The median PFS and median overall survival (OS) were 1172 days (range: 35‐2695) and 1396.5 days (range: 111‐2695) respectively. Advanced stage (III or IV) was 96.3% of HGSC, 13.3% of EC, 20.0% of CCC, 33.3% of MC, 75.0% of UC, and 33.3% of others (Table 1).

**Table 1:** Patient characteristics

	HGSC (n = 27)	EC (n = 15)	CCC (n = 30)	MC (n = 6)	UC (n = 4)	Others (immature teratoma 1, caricinosarcoma 3, SCC 1, mixed 1) (n = 6)	All (n = 88)
Age	55 (36‐85)	51 (34‐86)	50 (41‐77)	63.5 (48‐67)	58 (28‐65)	55.5 (37‐69)	55 (28‐86)
Stage	I: 0 II: 1 III: 20 IV: 6	I: 12 II: 1 III: 1 IV: 1	I: 23 II: 1 III: 6 IV: 0	I: 4 II: 0 III: 1 IV: 1	I: 1 II: 0 III: 2 IV: 1	I: 1 II: 3 III: 2 IV: 0	I: 41 II: 6 III: 32 IV: 9
Recurrence	Yes: 18 No: 9	Yes: 1 No: 14	Yes: 6 No: 24	Yes: 2 No: 4	Yes: 1 No: 3	Yes: 2 No: 4	Yes: 30 No: 58
PFS (Days)	712 (35‐2423)	1813 (494‐2695)	1545.5 (266‐2602)	960.5 (56‐1871)	2001 (256‐2690)	2086 (377‐2623)	1172 (35‐2695)
OS (Days)	895 (111‐2423)	1813 (594‐2695)	1562.5 (432‐2602)	960.5 (114‐1871)	2001 (760‐2690)	2086 (760‐2623)	1396.5 (111‐2695)

### 3.2 Actionable gene alterations and druggable gene alterations in malignant ovarian tumors

**Figure 1:**
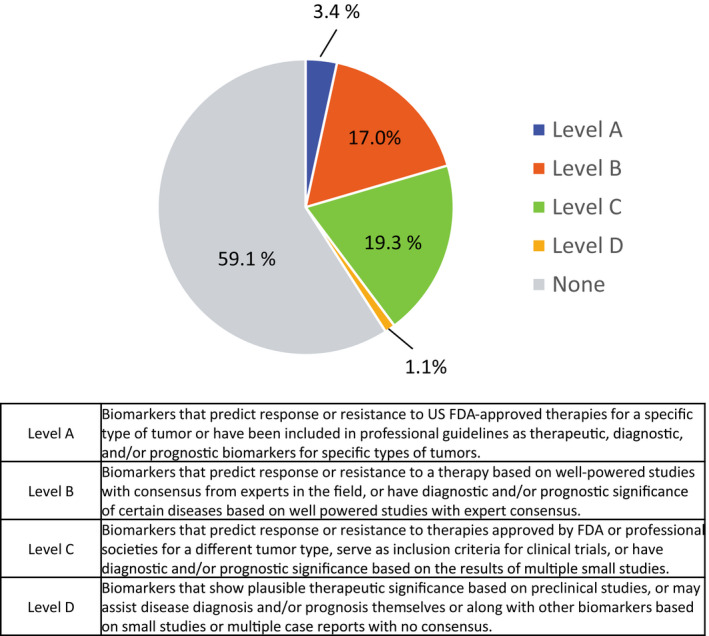
Categories of drug recommendation levels for all patients

We successfully sequenced genomic DNA from 88 patients with a mean sequencing depth of 978.5x (717x–1352x). Actionable gene alterations were identified in 80 of 88 samples (90.9%): HGSC, 92.6%; EC, 100.0%; CCC, 90.0%; MC, 83.3%; UC, 75.0%; and others, 83.3% (Table 2). Druggable gene alterations were identified in 36 of 88 samples (40.9%): HGSC, 14.8%; EC, 66.7%; CCC, 43.3%; MC, 66.7%; UC, 25.0%; and others, 66.7% (Table 2). Among the patients, 3.4% were identified for Level A drug recommendation, 17.0% were identified for Level B drug, 19.3% were identified for Level C drug, 1.1% were identified for Level D drug, and 59.1% had no drug recommendation (Figure 1).^8^

**Table 2:** Actionable and druggable alterations identified

.	HGSC (n = 27)	EC (n = 15)	CCC (n = 30)	MC (n = 6)	UC (n = 4)	Others (n = 6)	Total (n = 88)
Actionable alterations							
Yes (n (%))	25 (92.6%)	15 (100.0%)	27 (90.0%)	5 (83.3%)	3 (75.0%)	5 (83.3%)	80 (90.9%)
No (n (%))	2 (7.4%)	0 (0.0%)	3 (10.0%)	1 (16.7%)	1 (25.0%)	1 (16.7%)	8 (9.1%)
Druggable alterations							
Yes (n (%))	4 (14.8%)	10 (66.7%)	13 (43.3%)	4 (66.7%)	1 (25.0%)	4 (66.7%)	36 (40.9%)
No (n (%))	23 (85.2%)	5 (33.3%)	17 (56.7%)	2 (33.3%)	3 (75.0%)	2 (33.3%)	52 (59.1%)

**Figure 2:**
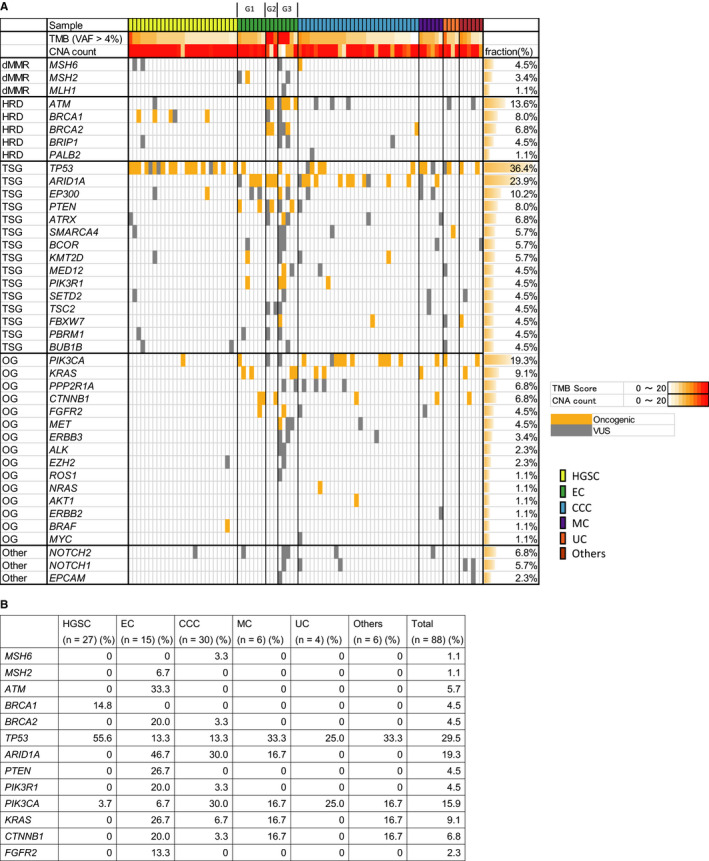
Actionable gene variants identified in malignant ovarian tumors. Oncogenic variant and variant of unknown significance (VUS) are depicted (A). List of extracted actionable gene variants in malignant ovarian tumors (B). TMB: tumor mutation burden; VAF: variant allele frequency; CNA: copy number alteration; dMMR: deficient of mismatch repair; HRD: homologous recombination deficiency; TSG: tumor suppressor gene; OG: oncogene; HGSC: high‐grade serous carcinoma; EC: endometrioid carcinoma; CCC: clear cell carcinoma; MC: mucinous carcinoma; UC: undifferentiated carcinoma

Regarding the actionable gene variants in mismatch repair (MMR) genes, the *MSH6* actionable gene variant was identified in 3.3% of CCC and *MSH2* was identified in 6.7% of EC (Figure 2A, B). Actionable *MLH1* or *PMS2* gene variant was not observed in all the subtypes analyzed. For the genes responsible for HRD, *ATM* actionable gene variant was identified in 33.3% of EC, *BRCA1* was identified in 14.8% of HGSC, and *BRCA2* was identified in 20.0% of EC and 3.3% of CCC. *BRIP1* and *PALB2* actionable gene variants were not identified; however, a variant of unknown significance (VUS) was found in some cases (Figure 2A, B).

*TP53* actionable gene variant was identified in 55.6% of HGSC, 13.3% of EC, 13.3% of CCC, 33.3% of MC, 25.0% of UC, and 33.3% of others. If VUS was included, *TP53* alteration was identified in 66.7% of HGSC. *ARID1A* actionable gene variant was identified in 0% of HGSC, 46.7% of EC, 30.0% of CCC, 16.7% of MC, 0% of UC, and 0% of others. *PTEN* actionable gene variant was identified only in EC (26.7%). *PIK3CA* actionable gene variant was identified in 3.7% of HGSC, 6.7% of EC, 30.0% of CCC, 16.7% of MC, 25.0% of UC, and 16.7% of others. *KRAS* actionable gene variant was identified in 26.7% of EC, 6.7% of CCC, 16.7% MC, and 16.7% of others. *FGFR2* actionable gene variant was identified only in EC (13.3%) (Figure 2A, B).

**Figure 3:**
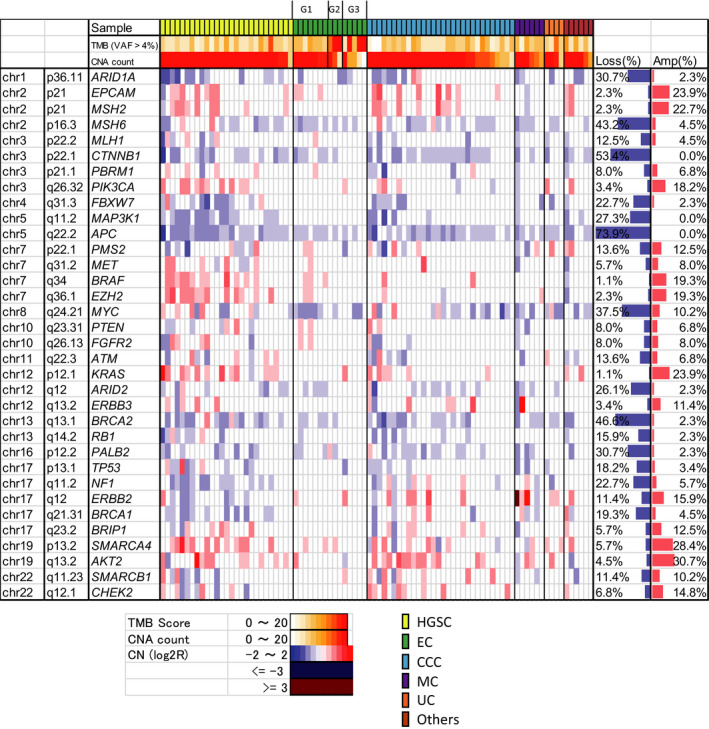
Copy number alterations (CNA) identified in malignant ovarian tumors. TMB: tumor mutation burden; VAF: variant allele frequency; CNA: copy number alteration; chr: chromosome; amp: amplification; CN: copy number; HGSC: high‐grade serous carcinoma; EC: endometrioid carcinoma; CCC: clear cell carcinoma; MC: mucinous carcinoma; UC: undifferentiated carcinoma

CNV showed frequent loss of *MSH6* (43.2%), *CTNNB1* (53.4%), *APC* (73.9%), and *BRCA2* (46.6%) (Figure 3). *ERBB3* amplification was identified in 11.4% of cases, of which 1 MC case showed CN = 12.5. Druggable *ERBB3* amplification was identified in 16.7% of MC (Figure 3, Table 3). *ERBB2* amplification was identified in 15.9% of cases, of which 1 MC case showed CN = 8.6 and 1 EC case showed CN = 38.0. Druggable *ERBB2* amplification was identified in 33.3% of MC (Figure 3, Table 3). *SMARCA4 (BRG1)* amplification was identified in 28.4% of the overall cases (Figure 3). CNV of 160 genes showed frequent gain of genes in 3q, 6p, 7q, and 12p and frequent loss in 6q, 9q, 13q, and 17q in HGSC, which was concordant with The Cancer Genome Atlas (TCGA) data^9^ (Figure S1). Focal loss of *NF1* was identified in 48.1% of HGSC (Figure 3), which was also reported in TCGA as focal deletion. *KRAS* was amplified in 44.4% of HGSC and 20% of CCC (Figure 3).

**Table 3:** Druggable alterations in malignant ovarian tumors

	HGSC (n = 27) (n (%))	EC (n = 15) (n (%))	CCC (n = 30) (n (%))	MC (n = 6) (n (%))	UC (n = 4) (n (%))	Others (n = 6) (n (%))	Total (n = 88) (n (%))	Drugs (Level of recommendation)
Hypermutation	2 (7.4%)	6 (40%)	3 (10.0%)	0 (0%)	1 (25.0%)	2 (33.3%)	14 (15.9%)	ICI (B)
Ultramutation	0 (0%)	1 (6.7%)	0 (0%)	0 (0%)	0 (0%)	0 (0%)	1 (1.1%)	ICI (B)
*BRCA1*	1 (3.7%)	0 (0%)	0 (0%)	0 (0%)	0 (0%)	0 (0%)	1 (1.1%)	PARP inhibitor (A), Platinum (B)
*BRCA2*	0 (0%)	0 (0%)	1 (3.3%)	0 (0%)	0 (0%)	0 (0%)	1 (1.1%)	PARP inhibitor (A), Platinum (B)
*PIK3CA*	0 (0%)	1 (6.7%)	8 (26.7%)	1 (16.7%)	1 (25.0%)	1 (16.7%)	12 (13.6%)	mTOR inhibitor (C), PI3K inhibitor (C), AKT inhibitor (C)
*CTNNB1*	0 (0%)	3 (20.0%)	1 (3.3%)	1 (16.7%)	0 (0%)	1 (16.7%)	6 (6.8%)	Imatinib (C), CWP232291 (C)
*MSH2*	0 (0%)	1 (6.7%)	0 (0%)	0 (0%)	0 (0%)	0 (0%)	1 (1.1%)	ICI (A)
*FGFR2*	0 (0%)	1 (6.7%)	0 (0%)	0 (0%)	0 (0%)	0 (0%)	1 (1.1%)	FGFR inhibitor (D)
*PTEN*	0 (0%)	1 (6.7%)	0 (0%)	0 (0%)	0 (0%)	0 (0%)	1 (1.1%)	mTOR inhibitor (C), AKT inhibitor (3A)
*AKT1*	0 (0%)	0 (0%)	1 (3.3%)	0 (0%)	0 (0%)	0 (0%)	1 (1.1%)	mTOR inhibitor (C), AKT inhibitor (C)
*AKT2* amplification	1 (3.7%)	0 (0%)	0 (0%)	0 (0%)	0 (0%)	0 (0%)	1 (1.1%)	mTOR inhibitor (C), AKT inhibitor (C)
*MET* amplification	0 (0%)	0 (0%)	1 (3.3%)	0 (0%)	0 (0%)	0 (0%)	1 (1.1%)	MET inhibitor (C)
*ERBB2* amplification	0 (0%)	0 (0%)	0 (0%)	2 (33.3%)	0 (0%)	0 (0%)	2 (2.3%)	HER2 inhibitor (C)
*ERBB3* amplification	0 (0%)	0 (0%)	0 (0%)	1 (16.7%)	0 (0%)	0 (0%)	1 (1.1%)	U3‐1402 (D)

Categories of drug recommendation levels are depicted in Figure 1. ICI, immune checkpoint inhibitors.

Druggable gene alterations were identified in 40.9% of the cases. Among them, hypermutation was found in 7.4% of HGSC, 40.0% of EC, 10.0% of CCC, 0% of MC, 25.0% of UC, and 33.3% of others; while ultramutation was found only in EC (6.7%). Druggable *BRCA1* alteration was found in only HGSC (3.7%), and druggable *BRCA2* variant was found in only CCC (3.3%). For the tumor suppressor genes, variants were not considered druggable if loss of heterogeneity or uniparental disomy did not occur. Druggable *PIK3CA* variant was identified in 0% of HGSC, 6.7% of EC, 26.7% of CCC, 16.7% of MC, 25.0% of UC, and 16.7% of others. Druggable *MSH2* variant was found in only 1 case of EC (6.7%). Druggable *FGFR2* gene variant was identified in 6.7% of EC *AKT2* amplification was identified in 3.7% of HGSC, and *MET* amplification was identified in 3.3% of CCC (Table 3).

### 3.3 TMB and CNA of malignant ovarian tumors

**Figure 4:**
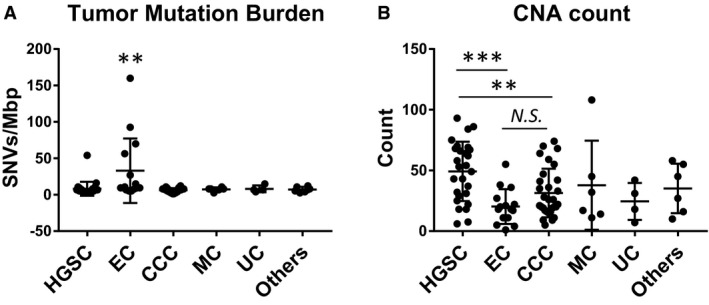
Tumor mutation burden (TMB) of malignant ovarian tumors. SNVs/Mbp. **, *P* < .01. (A). Copy number alteration (CNA) count of malignant ovarian tumors. **, *P* < .01; ***, *P* < .005; *N.S.*: not significant. (B). HGSC: high‐grade serous carcinoma; EC: endometrioid carcinoma; CCC: clear cell carcinoma; MC: mucinous carcinoma; UC: undifferentiated carcinoma

**Figure 5:**
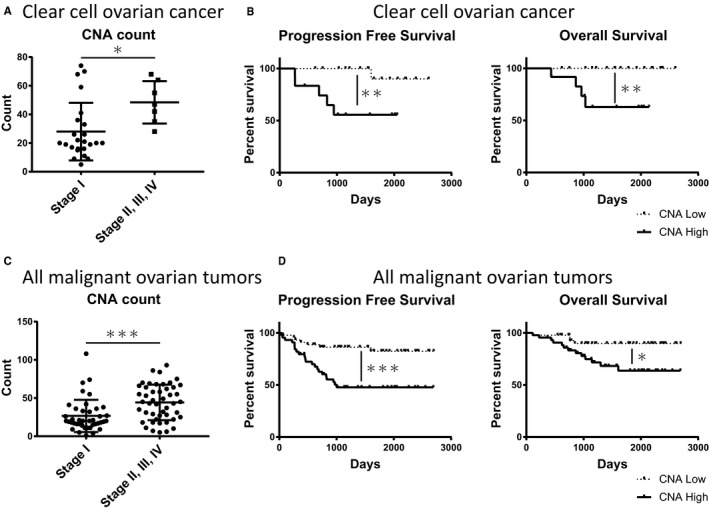
Copy number alteration (CNA) count of clear cell ovarian cancers in stage I and advanced stage (II, III, IV). *, *P* < .05. (A). Progression‐free survival (PFS) and overall survival (OS) analysis in CNA high (CNA count ≥ 30) and CNA low (CNA count < 30) clear cell ovarian cancers. **, *P* < .01. (B). CNA count of all malignant ovarian tumors in stage I and advanced stage (II, III, IV). ***, *P* < .005. (C). PFS and OS analysis of CNA high (CNA count ≥ 30) and CNA low (CNA count < 30) for all malignant ovarian tumors. *, *P* < .05; ***, *P* < .005. (D)

The median TMB was as follows: HGSC, 6.7 (range 2.7‐54); EC, 9.4 (5.4‐159.8); CCC, 6.7 (1.3‐12.1); MC, 8.1 (2.7‐10.7); UC, 6.7 (4‐14.8); and others, 6.75 (2.7‐12.1). EC had significantly higher TMB than the other histologic subtypes (*P* = .0012, one‐way ANOVA) (Figure 4A). The median CNA count was as follows: HGSC, 53 (range: 6‐93); EC, 18 (1‐55); CCC, 24 (5‐74); MC, 24.5 (11‐108); UC, 24.5 (7‐42); and others, 36 (10‐58). HGSC had significantly higher CNA count than the other tumor types (*P* = .0028, one‐way ANOVA) (Figure 4B). CNAs ≥ 40 were identified in 63.0% (17/27) of HGSC, 6.7% (1/15) of EC, 30% (9/30) of CCC, 33.3% (2/6) of MC, 25.0% (1/4) of UC, and 50% (3/6) of others. In CCC, advanced stage (II, III, or IV) was significantly correlated with high CNA count (*P* = .0187, t‐test) (Figure 5A), and high CNA count (CNAs ≥ 30) CCC tumors showed worse survival (PFS: Hazard Ratio (HR) 0.10, *P* = .0087; OS: HR 0.06, *P* = .0078, log‐rank test) (Figure 5B). In addition, the advanced stage (II, III, or IV) was correlated with high CNA count (*P* = .0004, t‐test) when all malignant ovarian tumors were included in the analysis (Figure 5C). High CNA count was correlated with worse survival in all malignant tumors (PFS: HR 0.28, *P* = .0007; OS: HR 0.30, *P* = .0146) (Figure 5D). In HGSC, high CNA count was insignificantly correlated with worse survival (*P* = .16 for PFS, *P* = .14 for OS) (Figure S2). In EC and MC, high CNA count was not related to survival, possibly due to the low rate of relapse and an insignificant number of cases analyzed (Figure S2).

## 4 DISCUSSION

Paclitaxel and carboplatin (platinum) combination therapy is primarily used for ovarian cancers regardless of the histologic subtype. However, advanced CCC and MC are resistant to standard platinum combination regimens compared with HGSC or EC.^10^, ^11^ Even in the same pathological diagnosis of ovarian cancer, genomic alterations are shown to be different.^9^, ^12^ Thus, treating ovarian cancer patients based on genomic biomarkers is necessary to improve outcomes. In our study using targeted capture sequencing of 160 cancer‐related genes, we found that 40.9% of malignant ovarian tumors had druggable alterations; this is similar to the result of MSK‐IMPACT in which clinically actionable gene alteration was detected in 37% of solid tumors.^5^

Actionable variants in MMR genes (*MLH1*, *MSH2*, *MSH6,* and *PMS2*) were not common in ovarian cancer. Only 1 CCC (3.3%) had *MSH6* variant, and only 1 EC (6.7%) had *MSH2* variant. Overall, in our study, 2.3% of malignant ovarian tumors harbored MMR gene variant (excluding VUS), which is almost concordant with a previous report that ovarian cancer has 1.3%–5% of MMR gene variant.^13^, ^14^ Actionable *ATM* gene variant was found in 33.3% of EC and was not detected in other histological types. *ATM* germline variant was found in only 0.9% of ovarian cancers^15^; our study indicates that *ATM* somatic variant may be common in EC. Actionable *BRCA1* variant was found in only HGSC (14.8%), while actionable *BRCA2* variant was found in 20.0% of EC and 3.3% of CCC. It was reported that 8.9% and 3.0% of ovarian cancer have *BRCA1* and *BRCA2* somatic variants, respectively.^16^ Sugino *et al* reported *BRCA1 or BRCA2 (BRCA1/2)* somatic variants in 5% of both CCC and EC.^13^ Our study also revealed that *BRCA2* somatic variant might contribute to the development of nonserous ovarian cancer except MC.

TCGA showed high CNAs, which is a hallmark of HGSC.^9^ In the present study, CNA count ≥ 40 was identified in 63.0% of HGSC and 30.0% of CCC, indicating that some populations in CCC have high CNAs. CCC with high CNA correlated with advanced‐stage tumor and worse survival, this was consistent with previous findings that CNA‐high breast and prostate tumors indicate worse survival.^17^, ^18^ One comparative genomic hybridization analysis of CCC found that amplification in 3 of the 4 chromosomal regions (8p11.21, 8p11.22, 12p13.31, and 20q13.2) had shorter OS.^19^ Our findings revealed that CNAs across the genome might be biomarkers of malignant ovarian tumors, including CCC. In addition, we found that the clinical target sequencing of 160 genes effectively allows the calculation of the CNA count, which can be a prognostic biomarker. PARP inhibitor was shown to be effective, especially in ovarian cancer with HRD or *BRCA1/2* mutation.^20^, ^21^ These clinical trials mainly targeted HGSC and EC. It has not been clarified if PARP inhibitors are also effective in treating CCC or MC. As CNAs can be considered a reflection of HRD, these CNA high CCCs may be treatable with PARP inhibitors. Germline or somatic HRD gene mutation was identified in 44% of HGSC, 28% of CCC, 23% of EC, and 16% of MC.^13^

SWItch/sucrose non‐fermentable (SWI/SNF) gene variants are common in endometriosis‐associated ovarian cancer, including CCC and EC. We found actionable *ARID1A* variant in 46.7% of EC and 30.0% of CCC, which is consistent with previous findings.^22^ *PIK3CA* actionable variant was identified in 3.7% of HGSC, 6.7% of EC, 30.0% of CCC, 16.7% of MC, 25.0% of UC, and 16.7% of others. It was reported that 2.3% of HGSC, 20.0% of EC, 20.0% of CCC, 0% of MC, and 7.1% of UC had *PIK3CA* variant.^23^ For EC, 61% have mutations in the PI3K pathway genes, including *PIK3CA*, *PTEN*, *PIK3R1*, *PIK3R2*, and *AKT1*.^24^ Our results confirmed that *PIK3CA* is druggable in certain populations and mostly targetable in nonserous ovarian cancer.

*KRAS* actionable mutation was found in 0% of HGSC, 26.7% of EC, 6.7% of CCC, 16.7% of MC, 0% of UC, and 16.7% of others. It was reported that 79.0% of MC and 0.6% of HGSC have *KRAS* mutation.^25^ Recently, *KRAS* mutation was found in 42% of EC.^24^ Although *KRAS* is the most frequently mutated oncogene in human cancers, no therapeutic agent directly targeting RAS has yet been approved. Our results show that *KRAS* was amplified in 44.4% of HGSC, and it was reported to be significantly locally amplified in TCGA.^9^ The development of KRAS inhibitor is under progress^26^, ^27^; thus, targeting KRAS will be a future option for ovarian cancer.

Druggable *ERBB2* amplification was found in 33.3% of MC, and druggable *ERBB3* amplification was found in 16.7% of MC. In MC, 19% were reported to have *ERBB2* amplification^28^; thus, HER2 inhibitors, such as HER2 ADC (trastuzumab‐deruxtecan), may be effective for such tumors. Our results indicate that targeting HER2 (*ERBB2)* or HER3 (*ERBB3*) can be considered in certain populations of MC. Druggable *MET* amplification was found only in 3.3% of CCC. MET inhibitor showed high response rate (50%) in a genomic matched trial of gastric cancer, which indicates that MET inhibitor can be considered for tumors with MET amplification.^29^

Hypermutation or ultramutation was found in 7.4% of HGSC, 46.7% of EC, 10.0% of CCC, 0% of MC, 25.0% of UC, and 33.3% of others. Although microsatellite instability‐high (MSI‐H) tumors are rarely seen in ovarian cancer, TMB indicates that about 50% of EC and 10% of HGSC or CCC can be treated with immune checkpoint blockade. A clinical trial on urothelial carcinoma revealed that TMB is associated with response rate to a greater extent than with PD‐L1 expression.^30^

Overall, targeted capture‐based genomic sequencing identified druggable alterations in about 40% of malignant ovarian tumors. It was reported that patients who received genomically matched therapy have longer PFS than the previous PFS^3^; thus, genomic testing can be applied in clinical settings to provide treatment strategies for malignant ovarian tumors. Our findings also demonstrate that CNA count could be a useful marker in clinical settings for risk assessment of patients with malignant ovarian tumors.

## CONFLICT OF INTEREST

ST, SN, and CO are employees of Mitsubishi Space Software Co., Ltd. All other authors declare no conflict of interest regarding this manuscript.

## AUTHOR CONTRIBUTION

KS carried out experiments, performed data analysis, and wrote the manuscript. TC contributed to the study design, experiments, results interpretation, and manuscript writing. EA, KN, HH, and HN contributed to the interpretation of results and data analysis. ST, SN, and CO performed data analysis. YK, HN, NS, TI, WY, FK, and DA contributed the study design and sample collection. All authors reviewed and collected the manuscript.

## Supporting information

Fig S1

Fig S2

Table S1

## Data Availability

The datasets generated/analyzed during the current study are available from the corresponding author on reasonable request.

## References

[1] Vaughan S , Coward JI , Bast RC , et al. Rethinking ovarian cancer: recommendations for improving outcomes. Nat Rev Cancer. 2011;11(10):719‐725. 21941283 10.1038/nrc3144 PMC3380637

[2] Sunami K , Ichikawa H , Kubo T , et al. Feasibility and utility of a panel testing for 114 cancer‐associated genes in a clinical setting: a hospital‐based study. Cancer Sci. 2019;110(4):1480‐1490. 30742731 10.1111/cas.13969 PMC6447843

[3] Dalton WB , Forde PM , Kang H , et al. Personalized medicine in the oncology clinic: implementation and outcomes of the johns hopkins molecular tumor board. JCO Precis Oncol. 2017;2017(1):1‐19. 10.1200/PO.16.00046 PMC6039131 30003184

[4] Hayashi H , Tanishima S , Fujii K , et al. Genomic testing for pancreatic cancer in clinical practice as real‐world evidence. Pancreatology. 2018;18(6):647‐654. 30055942 10.1016/j.pan.2018.07.006

[5] Zehir A , Benayed R , Shah RH , et al. Mutational landscape of metastatic cancer revealed from prospective clinical sequencing of 10,000 patients. Nat Med. 2017;23(6):703‐713. 28481359 10.1038/nm.4333 PMC5461196

[6] Hainsworth JD , Meric‐Bernstam F , Swanton C , et al. Targeted therapy for advanced solid tumors on the basis of molecular profiles: results from mypathway, an open‐label, phase IIa multiple basket study. J Clin Oncol. 2018;36(6):536‐542. 29320312 10.1200/JCO.2017.75.3780

[7] Tsumura K , Arai E , Tian Y , et al. Establishment of permutation for cancer risk estimation in the urothelium based on genome‐wide DNA methylation analysis. Carcinogenesis. 2019;40(11):1308‐1319. 31241739 10.1093/carcin/bgz112

[8] Li MM , Datto M , Duncavage EJ , et al. Standards and guidelines for the interpretation and reporting of sequence variants in cancer: a joint consensus recommendation of the association for molecular pathology, American society of clinical oncology, and college of American pathologists. J Mol Diagn. 2017;19(1):4‐23. 27993330 10.1016/j.jmoldx.2016.10.002 PMC5707196

[9] Integrated genomic analyses of ovarian carcinoma . Nature. 2011;474:609‐615. 21720365 10.1038/nature10166 PMC3163504

[10] Sugiyama T , Kamura T , Kigawa J , et al. Clinical characteristics of clear cell carcinoma of the ovary: a distinct histologic type with poor prognosis and resistance to platinum‐based chemotherapy. Cancer. 2000;88(11):2584‐2589. 10861437

[11] Perren TJ . Mucinous epithelial ovarian carcinoma. Ann Oncol. 2016;27(Suppl 1):i53‐i57. 27141073 10.1093/annonc/mdw087

[12] Itamochi H , Oishi T , Oumi N , et al. Whole‐genome sequencing revealed novel prognostic biomarkers and promising targets for therapy of ovarian clear cell carcinoma. Br J Cancer. 2017;117(5):717‐724. 28728166 10.1038/bjc.2017.228 PMC5572180

[13] Sugino K , Tamura R , Nakaoka H , et al. Germline and somatic mutations of homologous recombination‐associated genes in Japanese ovarian cancer patients. Sci Rep. 2019;9(1):17808. 31780705 10.1038/s41598-019-54116-y PMC6882827

[14] Song H , Cicek MS , Dicks E , et al. The contribution of deleterious germline mutations in BRCA1, BRCA2 and the mismatch repair genes to ovarian cancer in the population. Hum Mol Genet. 2014;23(17):4703‐4709. 24728189 10.1093/hmg/ddu172 PMC4119409

[15] Hirasawa A , Imoto I , Naruto T , et al. Prevalence of pathogenic germline variants detected by multigene sequencing in unselected Japanese patients with ovarian cancer. Oncotarget. 2017;8(68):112258‐112267. 29348823 10.18632/oncotarget.22733 PMC5762508

[16] Hennessy BT , Timms KM , Carey MS , et al. Somatic mutations in BRCA1 and BRCA2 could expand the number of patients that benefit from poly (ADP ribose) polymerase inhibitors in ovarian cancer. J Clin Oncol. 2010;28(22):3570‐3576. 20606085 10.1200/JCO.2009.27.2997 PMC2917312

[17] Zhang L , Feizi N , Chi C , Hu P . Association analysis of somatic copy number alteration burden with breast cancer survival. Front Genet. 2018;9:421‐421. 30337938 10.3389/fgene.2018.00421 PMC6178888

[18] Hieronymus H , Murali R , Tin A , et al. Tumor copy number alteration burden is a pan‐cancer prognostic factor associated with recurrence and death. eLife. 2018;7. 10.7554/eLife.37294 PMC6145837 30178746

[19] Morikawa A , Hayashi T , Kobayashi M , et al. Somatic copy number alterations have prognostic impact in patients with ovarian clear cell carcinoma. Oncol Rep. 2018;40(1):309‐318. 29749539 10.3892/or.2018.6419

[20] Moore K , Colombo N , Scambia G , et al. Maintenance olaparib in patients with newly diagnosed advanced ovarian cancer. N Engl J Med. 2018;379(26):2495‐2505. 30345884 10.1056/NEJMoa1810858

[21] González‐Martín A , Pothuri B , Vergote I , et al. Niraparib in patients with newly diagnosed advanced ovarian cancer. N Engl J Med. 2019;381(25):2391‐2402. 31562799 10.1056/NEJMoa1910962

[22] Wiegand KC , Shah SP , Al‐Agha OM , et al. ARID1A mutations in endometriosis‐associated ovarian carcinomas. N Engl J Med. 2010;363(16):1532‐1543. 20942669 10.1056/NEJMoa1008433 PMC2976679

[23] Campbell IG , Russell SE , Choong DY , et al. Mutation of the PIK3CA gene in ovarian and breast cancer. Cancer Res. 2004;64(21):7678‐7681. 15520168 10.1158/0008-5472.CAN-04-2933

[24] Cybulska P , Paula ADC , Tseng J , et al. Molecular profiling and molecular classification of endometrioid ovarian carcinomas. Gynecol Oncol. 2019;154(3):516‐523. 31340883 10.1016/j.ygyno.2019.07.012 PMC6736779

[25] Mueller JJ , Schlappe BA , Kumar R , et al. Massively parallel sequencing analysis of mucinous ovarian carcinomas: genomic profiling and differential diagnoses. Gynecol Oncol. 2018;150(1):127‐135. 29793804 10.1016/j.ygyno.2018.05.008 PMC6105459

[26] Kessler D , Gmachl M , Mantoulidis A , et al. Drugging an undruggable pocket on KRAS. Proc Natl Acad Sci USA. 2019;116(32):15823‐15829. 31332011 10.1073/pnas.1904529116 PMC6689897

[27] Janes MR , Zhang J , Li L‐S , et al. Targeting KRAS mutant cancers with a covalent G12C‐specific inhibitor. Cell. 2018;172(3):578‐589.e17. 29373830 10.1016/j.cell.2018.01.006

[28] Mishra R , Hanker AB , Garrett JT . Genomic alterations of ERBB receptors in cancer: clinical implications. Oncotarget. 2017;8(69):114371‐114392. 29371993 10.18632/oncotarget.22825 PMC5768410

[29] Lee J , Kim ST , Kim K , et al. Tumor genomic profiling guides patients with metastatic gastric cancer to targeted treatment: The VIKTORY umbrella trial. Cancer Discov. 2019;9(10):1388‐1405. 31315834 10.1158/2159-8290.CD-19-0442

[30] Rosenberg JE , Hoffman‐Censits J , Powles T , et al. Atezolizumab in patients with locally advanced and metastatic urothelial carcinoma who have progressed following treatment with platinum‐based chemotherapy: a single‐arm, multicentre, phase 2 trial. Lancet. 2016;387(10031):1909‐1920. 26952546 10.1016/S0140-6736(16)00561-4 PMC5480242

